# A case study of nurse practitioner role implementation in primary care: what happens when new roles are introduced?

**DOI:** 10.1186/1472-6955-12-1

**Published:** 2013-01-23

**Authors:** Esther Sangster-Gormley, Ruth Martin-Misener, Fred Burge

**Affiliations:** 1 School of Nursing, University of Victoria, PO Box 1700, STN CSC, Victoria, British Columbia, V8W 2Y2, Canada; 2 Dalhousie University School of Nursing, Box 15000, 5869 University Ave.Halifax, Nova Scotia, B3H 4R2, Canada; 3 Dalhousie University Dalhousie Family Medicine, AJLB 8 QEII HSC, 5909 Veteran's Memorial Lane, Halifax, Nova Scotia, B3H 2E2, Canada

**Keywords:** Nurse practitioner, Implementation, Role, Barriers, Facilitators, Role implementation, Primary care

## Abstract

**Background:**

At the time of this study (2009) the role of the nurse practitioner was new to the province of British Columbia. The provincial government gave the responsibility for implementing the role to health authorities. Managers of health authorities, many of whom were unfamiliar with the role, were responsible for identifying the need for the NP role, determining how the NP would function, and gaining team members’ acceptance for the new role.

**Method:**

The purpose of the study was to explain the process of nurse practitioner role implementation as it was occurring and to identify factors that could enhance the implementation process. An explanatory, single case study with embedded units of analysis was used. The technique of explanation building was used in data analysis. Three primary health care settings in one health authority in British Columbia were purposively selected. Data sources included semi-structured interviews with participants (n=16) and key documents.

**Results:**

The results demonstrate the complexity of implementing a new role in settings unfamiliar with it. The findings suggest that early in the implementation process and after the nurse practitioner was hired, team members needed to clarify intentions for the role and they looked to senior health authority managers for assistance. Acceptance of the nurse practitioner was facilitated by team members’ prior knowledge of either the role or the individual nurse practitioner. Community health care providers needed to be involved in the implementation process and their acceptance developed as they gained knowledge and understanding of the role.

**Conclusion:**

The findings suggest that the interconnectedness of the concepts of intention, involvement and acceptance influences the implementation process and how the nurse practitioner is able to function in the setting. Without any one of the three concepts not only is implementation difficult, but it is also challenging for the nurse practitioner to fulfill role expectations. Implications for research, policy, practice and education are discussed.

## Background

Advanced practice nursing is an umbrella term used to designate nursing practice that demonstrates competencies beyond the traditional scope of the registered nurse [1]. Interest in advanced practice nursing roles continues to spread around the world as evidenced by the 60 different countries in which such roles have been implemented [2-6]. While international interest continues to grow, there is no consensus on how best to define, introduce, or implement these roles in primary health care (PHC) [5,7,8]. The lack of consensus on how to implement advanced nursing roles has contributed to the complexity in establishing best practices for their implementation [2,7]. Consequently, there is a need for continued knowledge development of factors affecting successful role implementation.

The International Council of Nursing, International Nurse Practitioner/Advanced Practice Nursing Network (INP/APPN) defines nurse practitioner (NP) as “a registered nurse who has acquired the expert knowledge base, complex decision-making skills and clinical competencies for expanded practice, the characteristics of which are shaped by the context and/or country in which s/he is credentialed to practice” [9]. Although NPs have been in existence in the United States for more than 40 years, their introduction is more recent in other countries including Canada. Researchers have identified difficulties with implementing the NP role in PHC settings where team members are unfamiliar with it [10,11]. The purpose of this study was to understand and explain the process used to implement the NP role into a health authority in BC, and factors that influenced the ability of NPs to enact the role. The purpose of this paper is to describe the results of that study.

In British Columbia (BC), Canada, legislation and regulation of the NP role were established in 2005 and the first NPs were hired into regional health authorities beginning in 2005. Health authorities in BC are funded by the Provincial Ministry of Health. NPs are salaried employees of health authorities. They practice in acute and long-term care and in PHC settings. This study was conducted in 2009 and, at that time, the NP role was relatively new to the province of BC, and there were less than 200 NPs registered in the Province [12].

Health Canada [13] defines PHC settings as the first place people go when they need care, advice on health promotion or illness prevention, and/or referral to other parts of the health care system, and where care is delivered to individuals, families, communities, and populations of patients. In the last 20 years numerous Canadian provincial and national reports have highlighted the need for changes to the way PHC is delivered [14-17]. Nationally identified concerns include inadequate attention to health promotion and disease prevention, lack of continuity of care among providers and institutions, difficulty obtaining access to care, barriers to integrating primary health care providers such as NPs into the system, and the need for intersectoral action and collaboration [14,15,18].

As part of PHC reform, federal and provincial governments have been interested in implementing the NP role in primary health care [15-17,19]. Consequently, between 1996 (Alberta) and 2009 (Yukon), all provinces and territories in Canada enacted legislation enabling the NP role [20]. The province of British Columbia (BC) was one of the last provinces to implement legislation for regulation of the role [21].

The health authority, included in this study, began implementing the NP role in 2007 with the hiring of the first NPs in PHC settings. NPs were expected to increase access to care, and manage chronic diseases. Senior health authority managers allocated responsibility for overseeing implementation to a newly established NP steering committee. The committee’s mandate was to develop an NP role description, create a format for submission of proposals for new NP positions, and recommend strategies to introduce and implement the new role.

### Implementation

What is meant by implementation can differ from one setting to another; therefore, in this study, we defined implementation as the process used by the health authority to add an NP to the health care team in PHC settings. From the literature we knew that complex organizational systems function synergistically to influence expected outcomes of any new initiative [22-25]. Implementing the NP role in a health care system does not occur in isolation of the overall system because contextual and environmental issues influence the process.

Canadian researchers have identified barriers to implementing the NP role in acute and long-term care, and PHC settings. Barriers include restrictive legislation and regulation of the role [10,11], lack of role descriptions and understanding of the role, conflicting expectations, inadequate administrative and physician support and insufficient long-term human resource planning, as well as poor understanding of how the NP role interfaces with other members of the health team [10,11,26-31].

Although multiple barriers to implementation have been identified, facilitators also exist. Facilitators include manager and physician support and knowledge of the NP role, the NP’s prior work experience and level of education, trust and acceptance by team members, and patient satisfaction with the NP [10,26-32]. Managers can facilitate implementation by helping team members to understand the reason for adding the role, by supporting them as they experience the stresses of change that occur with the addition of the new role, and by helping to incorporate the NP into the team [32].

Findings of previous researchers demonstrated that successfully implementing the NP role for the first time into PHC settings is a complex process. In our review of current literature, we found that facilitators in one setting could become barriers if they were not addressed appropriately, and likewise, barriers could become facilitators [33]. Clearly, these barriers or facilitators influence role implementation and the long-term sustainability of the role. Based on the work of previous researchers, we recognized the need to better understand NP role implementation, and to explore influencing factors more deeply.

Therefore, we began this study with an integrative review of the literature which resulted in identifying and defining three sensitizing concepts that influenced implementation [33]. The concepts were intention, involvement and acceptance. We defined intention as how the role is defined and the goals and outcomes expected as a result of implementation. Involvement refers to the active participation of team members in determining the functions for the NP role. Acceptance relates to the team members’ recognition and willingness to work with the NP. A complete description of the integrative review and the process used to develop the concepts has been reported elsewhere, thus we will not repeat the description in this paper [33]. The three concepts were subsequently used to develop the research questions that guided our study.

The research questions were:

1. How do intentions for the NP role identified in PHC settings influence the process of NP role implementation?

2. How are managers, physicians, other staff, and NPs involved in the process of NP role implementation in PHC settings within the health authority?

3. How does acceptance by managers, physicians, and other staff of the NP role in PHC settings influence the process of implementation?

4. How are NPs enacting the role domains of clinical practice, collaboration, research, leadership, and change agent in PHC settings?

### Ethics approval

This study received ethics approval from the Human Research Ethics Board for Dalhousie University, the University of Victoria, and the health authority in which the study was carried out (Protocol #2008-1896). Written informed consent was obtained from each participant before the interview began. Only adults 18 years of age and older were selected to participate.

## Methodology

### Design

To answer the research questions we used an explanatory single case study with three embedded units of analysis and adhered to Yin’s [34] approach for case study research. We also employed Yin’s technique of explanation building for data analysis. The goal of analysis using this technique is to build an explanation about the case, or to explain how the NP role was implemented in the health authority. In a narrative format, we built an explanation of the process by using an iterative process of comparing the data to the study’s conceptual framework and research questions [34]. By constantly referring to these, we were able to maintain our focus on how the role was implemented and how, or if, the concepts of intention, involvement and acceptance influenced the process. The case studied was the process of NP role implementation as it was occurring in PHC settings in one of BC’s six health authorities. We refer to the settings as: PHC 1, physician office; PHC 2, seniors’ care centre; and PHC 3, mental health care team. In each setting we followed the same research protocol, which meant posing the same interview questions to key informants in similar roles, and reviewing similar documents [34]. Written informed consent was obtained from each participant before the interview began. Only adults 18 years of age and older were selected to participate.

### Data Sources

In each PHC setting we interviewed the manager directly responsible for the administrative day-to-day functioning of the setting, at least one physician working with the NP, and at least one of the staff such as an RN or a medical office assistant, and the NP. In addition to interviews, we conducted document reviews to corroborate data from the interviews or contribute to our understanding of the context of the setting [34].

### Inclusion criteria

We only included settings where the NP had been working for a minimum of six months. Only English speaking participants, who worked directly or indirectly with the NP, and were working in the PHC setting six months or more before the NP was hired were included. Because these participants had been in the setting before and during the time the NP was hired, they provided unique perspectives and insights into how intentions for the role were developed, who was involved in the decision to hire the NP, and how acceptance for the role occurred [35].

### Data collection

All interviews, with the exception of one, occurred face-to-face in a private, mutually agreed upon location. One interview was conducted over the telephone. Interviews took approximately 60 minutes and were audio-recorded and later transcribed verbatim. One researcher (ESG) conducted all interviews and reviewed all documents. An interview guide was used to generate discussion of how key informants were involved in the implementation process, their understanding of the intention for the role, and their views on how the role was accepted (available upon request).

Pertinent documents such as project charters, the proposal submitted for approval of the NP position, and the NP role description were reviewed. We accessed information pertaining to the health authority, for example the strategic plan, from its website. We accessed municipal websites for information related to geographic locations of the settings. Competencies and standards for NP practice were obtained from the College of Registered Nurses of British Columbia’s website. Table 1 is a summary of the number and types of participants interviewed in each setting and documents reviewed.

**Table 1:** Data sources

**Participant interviews**	**PHC 1**	**PHC 2**	**PHC 3**
	**Physician office**	**Seniors care centre**	**Mental health care team**
Manager	2	1	1
Physician	1	1	1
RN	1	0	1
NP	1	2	1
Medical office assistant	0	1	0
Staff coordinator	0	1	0
Community member	0	0	1
**Total participants**	**5**	**6**	**5**
Documents Reviewed	Demonstration project charter	Project charter	Proposal
	NP role description, NP competencies and scope of practice, health authority’s strategic plan		

### Data analysis

Interview data were imported into N-Vivo 8.0. We began data analysis by first capturing data related to the concepts of intention, involvement and acceptance [36,37]. Second, within each concept, we sorted data into categories and themes. Third, as more data were collected, preliminary codes were expanded and collapsed to refine the coding categories [38]. Finally categories and relationships emerged from the data that was used to explain how the sensitizing concepts influenced implementation.

We used a data abstraction tool developed for this study to assist with examining the documents for content related to the NP role and how the NP was expected to function. Key documents were not coded but were used to understand the social context in which they were developed and supported the writing of the narrative describing how the NP role was implemented in each PHC setting. Because of the small sample size and the need to protect confidentiality, we refer to participants collectively and do not identify anyone by role, gender, or title.

### Validity and reliability

Validity and reliability were addressed in a variety of ways. Initially, we developed a case study protocol which included interview questions, and a list of the types of documents to select for review. Data were obtained from a variety of key informants and documents. At the completion of each interview, we summarized our understanding of informants’ responses and asked them to verify the accuracy of our summary. We did not return transcripts to participants for their review. Throughout the study we discussed our findings among ourselves and used rich, thick descriptions to explain our findings [34].

## Results

We used an explanatory single case study of one purposively selected health authority with three embedded sub-units of analysis (PHC settings) that were also purposively selected [39]. Variation among the settings included geographic location, model of care, and patient populations. Table 2 illustrates the model of care, population density and patient population of each unit of analysis.

**Table 2:** Units of analysis

**PHC Setting**	**PHC 1**	**PHC 2**	**PHC 3**
Model of Care	Physician office	Seniors PHC Centre	Mental Health Care Team
Location	Small urban	Urban	Rural/remote
Population density	>20,000	>100,000	3,800
Patient Population	Family practice	Seniors 70+	Mental health & addictions

From our data collection we learned that the decision to hire an NP in PHC settings was determined in one of two ways: 1) an administrative directive from senior health authority managers, and 2) approval of proposals developed and submitted to the NP steering committee by managers from PHC settings. Either way, the process did not require involvement of other team members in early discussions.

For example, the process used in the physician office (PHC 1) and the seniors care centre (PHC 2) involved senior health authority managers meeting with the physicians who indicated interest in the NP role and/or developing project proposals that were submitted to a federal government initiative called the Primary Health Care Transition Fund [40] for funding. Similarly, the managers who submitted proposals to the NP steering committee were not required to involve team members in the writing of the proposal. As a result of this top down approach to implementation, before the NP was hired, few team members participated in efforts to determine a need for an NP or how the addition of an NP would change care delivery.

This was significant because at the time most of the NPs in this study were hired (2007 & 2008) the role was new to BC, having been legislated in 2005. As a result, team members in PHC settings were unfamiliar with the role and had little knowledge of how best to use the NP’s capabilities. The first BC NPs, who graduated in 2005, had been mentored by family physicians during their educational programs because there were no registered NPs in the province until 2006. This meant that the first NP graduates had only been exposure to the NP role through NP faculty members teaching in the programs. Therefore, NPs were hired into settings where few, if any, team members were involved in hiring the NP, and where they had not participated in discussions of how the NP would function, the types of patients the NP care for, nor how the NP role interfaced with other roles in the settings.

### Intentions for NP Role implementation

Before the NP was hired, study participants were aware that the health authority intended for NPs to provide direct patient care for various populations such as elderly, or people with chronic diseases. However, for participants, knowing that NPs were expected to increase access to care for patients, improve patient outcomes, and the function of interdisciplinary teams did not provide insight into how an NP would be expected to enact the role or function within the existing team. In all three settings, after the NP was hired team members, including professional staff, physicians, other community providers, and managers had to work together to identify or clarify role expectations and determine how the NP would function as a new team member.

After the NP was hired, unexpected changes, such as the retirement of a physician in PHC 1 and increasing the age of the patient population in PHC 2, precipitated the need for participants to clarify how the NP would function. The original intent in PHC 1 was that the NP would co-manage patients with chronic diseases; however, after a physician unexpectedly retired, the team needed to reassign the physician’s patients to another provider. In PHC 2 shortly after the NP was hired, the age of new patients admitted to the centre increased from 55 years and older to 70 years and older. Many of the new older people were frail and had more complex care needs.

In both instances, team members approached senior health authority managers for assistance in understanding how to proceed with implementation. Because participants were unfamiliar with the NP role, they wanted more direction and structure from senior managers and looked to them for advice.

"I think that for any organization that is looking at employing a nurse practitioner there needs to be managerial support, logistical support, and practice support from the beginning. I can’t stress this enough. There is a need for more than getting the nurse practitioner’s office set up and computer access, those kinds of things were all fine. But when it came to the day to day types of things there needs to be a bit more support from upper management."

Senior health authority managers responded to these requests but the response took three months in PHC 2 and, because of turnover of health authority managers, six months in PHC 1. These delays slowed the teams’ discussions of the most appropriate patients for the NP to follow, made it difficult for team members to obtain mutual understandings of role functions, and, in PHC 2, contributed to turnover of NPs.

In addition to senior health authority managers, participants remarked that team members also looked to NPs to explain their role, and although new in the role, NPs assumed leadership in varying degrees to define it.

"[NP] certainly exhibited leadership with the team, helping the team understand the nurse practitioner role, showing leadership in terms of clinical competencies, and development and professional standards. [NP] also demonstrates leadership in looking at patient populations and helping the team consider how to deliver care differently."

The NP’s knowledge and understanding of the role and ability to explain it to others contributed to the team’s appreciation for how the NP would function. Ultimately, after the NP was hired, team members worked together to clarify the types of patients the NP would follow and how the NP would function within the team.

"I think that there may have been a little bit more expectation when it was set up that the nurse practitioner would deal with populations that the health authority, as an organization, had chosen as key populations that they felt had gaps in service. I think that changed as the nurse practitioner role developed in the clinic because the clinic had its own needs. So it developed according to the clinic’s populations and needs rather than what the health authority had seen as their populations. It was different in each clinic because the health authority was looking at a very large population and pockets of patients who may not be appropriate in one area."

The process of working together to clarify the intentions and expectations of how the NP would function in each setting to match the needs of the clinic evolved over time and required time commitments from team members. Because planning for the role took time and this work was not initiated before NPs were hired, it had to occur afterwards. During this time, while team members worked to define the role, the NPs were constrained in how they practiced.

### Defining the role

Participants were asked to define the NP role based on their experience working with the NP. All participants related most readily to the clinical aspects of the role, and believed the NP was additive to the team, a bonus. One participant defined the role broadly:

"The nurse practitioner is primarily a nurse who has expanded training in diagnosis and treatment. [NP’s] role on a day to day basis is to manage the care of patients that present to the clinic, whether they’re [NP’s] own patients or the clinic’s patients. [NP] manages episodic, simple primary care problems like a sore throat and complete physicals and chronic disease management and women’s health. Because as a nurse the nurse practitioner has the skills and training to look at the patient’s needs from more of a holistic, psycho-social as well as physical aspect [NP] can manage more fully the whole impact of the patient’s illness or wellness and also deal with the family."

"On the other hand when the patient becomes more complex medically, [NP] then knows when to turn the patient over or consult with a physician, whether it’s a one off consult, “what’s your opinion” or it’s a hand off consult, “this is beyond my scope of practice”. The nurse practitioner role is in primary care management of episodic illness, chronic diseases, promoting wellness, education of patients and their families, as well as contributing to planning for the communities’ needs from a health standpoint and providing some leadership with other members of the medical community including other health team members like physio [physiotherapist], OT [occupational therapist], other nurses, LPNs [Licensed Practice Nurse], clerical staff."

NPs in all three settings worked full-time and reported that they spent at least 75% of their time providing direct patient care. In PHC 1, participants recognized that the NP managed more complex patients and practiced differently than the RNs. They also believed that, as a salaried employee, the NP spent time with patients focusing on chronic disease management, health promotion, and self-care management. Similarly, participants in PHC 2 commented on the NP’s ability to see the “big picture”. They remarked that she identified patients in need of community resources and collaborated with all members of the team, as well as community agencies. The NP in PHC 3 was described by participants as the missing piece to the team and complemented the efforts of other team members to provide comprehensive patient care. Participants acknowledged that NPs spent more time with patients than physicians, and provided more patient education related to self-care management of health conditions, as this participant noted:

"I think in terms of the primary health care setting, they function sometimes better than the physicians. Because nurse practitioners are not in a fee-for-service agreement, they have more time to spend with each patient in terms of counseling services. Family physicians usually don’t have enough time to fully counsel patients with regards to the conditions. It sometimes takes a few visits to go over all the details family physicians want to go through. But with [NP] because of the freedom of time offers a better service than family physicians."

The NP was also viewed as a competent, knowledgeable provider who offered comprehensive patient care and who was an asset to the overall team. Participants related that, as they witnessed the NP’s practice and their knowledge and understanding of the NP role increased, they developed trust in the NP’s capabilities and saw the value of the role. The ability of team members to define the role within the context of the PHC setting required time and effort on the part of all team members and did not come easily.

### Finding space

In all settings, NPs expected that they would have a physical space in which to practice. A participant described the need to plan for space in which the NP would practice:

"It is important to prepare the site ahead of time. By preparing, I mean look at what we are doing now and what it is going to look like when an NP comes. Rather than appending the role to the existing team, it involves the re-creation of the whole team. Team development is important and it needs to begin before NPs are hired so there is a space for them when they come in."

In PHC 2 an office space and examination room was not identified for the NP until after she was hired. Similarly, in PHC 3, once the NP was hired, the team had to reassess the most appropriate place for the NP to practice. After consulting with community stakeholders, they determined that the best place for the NP to see patients was in a community resource centre. It took four months for that space to be identified. Lack of physical space was a barrier to the NPs’ practice because the NPs in PHC 2 and PHC 3 could not begin to practice until space was identified. A participant related the need to have designated space for the NP before the NP is hired:

"There is a need to get the system clearer upfront and have designated room for an NP. It is not fair if the nurse practitioner doesn’t have a decent examination room. The nurse practitioner is part of the team and everyone should have a working station. The nurse practitioner needs a room like physicians. They need to have an exam room with curtains and everything."

Participants thought there should have been a system in place for planning for the NP by designating physical space where the NP would practice before the NP was hired.

### Long-term planning

PHC 1 was established as a 24 month demonstration project, with no guarantee that the NP would be in the setting over the long term. All participants were aware of this, however, 18 months into the project, senior health authority managers had not communicated with the NP or others about any plans for the future of the project and the NP’s position. Without knowledge of the health authority’s plans, participants were unsure of the sustainability of the role, and this contributed to the NP’s decision not to admit any new patients until the future of the role was clarified. 

Reflecting on the implementation process, in all three settings participants identified the need to plan, in advance, for the addition of an NP, to identify expectations, and to identify appropriate space for where the NP would practice. One participant described it this way:

"What I would do differently? First of all, we still have a ways to go before we can take a package that says “all of these things have to be done in this order when the nurse practitioner starts” so that on the NP’s first day everything is in place. If the planning is done before the NP is hired, on the first day of work everybody will know what the role is. Any ground work would be done prior to the NP starting work."

Maybe better exploration of what the clinic thought the role would be. Although, sometimes it’s nice for them to evolve it on their own, because it makes the nurse practitioner, the office managers, all of the clericals and the physicians to work through a PDA (plan, do, act) cycle to see what works best. I’m not sure you can really mould the role beforehand. However, I think we need to make sure everybody is clear on what the potential for the role is and where nurse practitioners could be used. But I wouldn’t want to be too stringent on what we’re doing because I think it changes.

### Involvement of managers, physicians, and other staff in role implementation

Few team members were involved in the early stages of implementation, when plans were first made to hire an NP. Participants in PHC 1 and PHC 3 were aware that discussions were taking place or a proposal had been submitted, but they were not directly involved. In these two settings, participants indicated that this was not problematic for them. Yet, in PHC 2, the lack of team involvement in the decision of when to hire another NP after the first NP resigned was awkward. A participant remarked:

"We all knew from day one there would be an NP. So it wasn’t a surprise that another nurse practitioner was being hired. But the way in which the NP came in was not particularly clear and in hind sight that made it even harder. Somewhere along the way somebody’s communication went a bit astray."

Inadequate involvement created problems for participants in PHC 2. They related that they were less invested in the process because of their lack of involvement in the NP hiring plans.

In PHC 1 the NP mailed letters to community providers describing the role, however, once medical specialists received patient referrals from the NP or a patient presented prescriptions written by the NP to local pharmacies, these providers called the office asking for clarification of the NP’s role. Others in the office, such as the business manager and the RNs were involved in fielding these calls. In PHC 2 team members were involved in discussions of which patients to schedule appointments with the NP. In PHC 3 the manager and lead RN were involved in identifying community service agencies that provided services to the same population with whom the NP worked.

### Community involvement

In PHC 1 people living in the community and other providers were not consulted or advised of plans to hire an NP. The NP role was new in the community and, in the absence of early community involvement or an announcement; these stakeholders had no knowledge of the NP’s capabilities or presence in the community. In contrast, in PHC 3 discussions were held with physicians and members of community agencies involved with caring for people with mental health and addictions before the NP was hired. A participant from PHC 3 described the process used to include these stakeholder groups as:

"We pulled together groups of people that were related to people with profound mental health problems. We talked to hospital nurses, some of the local First Nations workers that have history of working with these people, the Friendship Centre, a few clergy people, and local doctors."

Although there was limited involvement of community stakeholders in each setting, their early involvement was important.

Moreover, in all three settings, patients in the practices were unaware of the NP role and participants acknowledged that they needed to encourage patients to schedule an appointment to meet and get to know the NP. Patients needed to trust the NP before they allowed the NP to provide their care, and there were times when patients were unwilling to schedule an appointment with the NP, as one participant described:

"Occasionally I would take a call from a patient and they would want a doctor and I would say “our nurse practitioner could handle your problem” and they would go “no I want a doctor.”"

### Manager involvement

Involvement of the managers in all three settings was critical. In general, managers supported novice NPs to identify space in which to work, obtain equipment and meet key stakeholders. Moreover, the managers in all three settings supported the NPs to participate in the community of practice, and facilitated discussions of how the NP could be used in the setting. A participant described the manager’s involvement:

"Well I think advocating for the role and trying to negotiate and understand where everyone was coming from because everybody has their own world to live in. Trying to figure out how to best meet everybody’s needs and still ensure that the patient gets the test and the nurse practitioner has the information needed to manage patients. And also advocating for the nurse practitioner role and paving the way for other nurse practitioners to come."

In the event managers were unable to answer team members’ questions or concerns related to how to use the NP, they looked to senior health authority managers who had been responsible for establishing strategic direction for NP role implementation to clarify organizational intent.

### Acceptance of NP role implementation

In the PHC settings, according to participants, team members needed time to become acquainted with the NP and gain a better understanding of the role before accepting the NP as a new team member. A participant described one way this was accomplished:

"We had a number of meetings with the nurse practitioner and the team, the nurse practitioner and the physicians, reviewing the standards, limits, and conditions of the role and all that sort of stuff and really trying to be clear about what the nurse practitioner could do."

As well, community providers, such as medical specialists, wanted to become acquainted with the NP’s competencies and scope of practice before their acceptance occurred.

Team members’ acceptance of the NP role was influenced by their involvement in clarifying the intentions for the role, their increased understanding of what the NP would do in the practice setting, and trusting in the NP’s capabilities. One participant noted, “Everyone needs to trust the nurse practitioner and if they trust that the nurse practitioner knows what he or she is doing, they’re a little more comfortable.” Other factors, such as prior knowledge of the individual NP, the NP’s personal attributes, and patient acceptance also contributed to the team’s willingness to work with the NP. In all three settings, the NP had previously spent time as a student NP in the practice setting or in the community in which the setting was located.

Prior knowledge and acquaintance with the NP, for example working in the setting as an RN or spending time in the setting as a student NP prior to being hired, allowed acceptance to develop more quickly as a participant described:

"[NP] had been working here as a nurse and has been on the team. We knew that [NP] was attending a nurse practitioner program and hoped to transition into a nurse practitioner role somewhere. When the position came up we were quite aware of the timing and honestly I think that we all hoped that [NP] would end up here. We knew [NP] and very much respected [NP’s] abilities and enjoyed [NP’s] personality."

Prior knowledge by team members of the NP as a student NP or as an RN, gave those team members an opportunity to establish a relationship, become aware of the NP’s capabilities, and develop trust. Various participants described NPs as knowledgeable with good communication skills. NPs also assumed leadership roles by working collaboratively with other team members to help them understand the role which also facilitated team acceptance.

Patients cared for by the mental health care team (PHC 3) typically did not readily accept new health care providers. It took time for patients in this setting to trust the NP. This was also true in the other two settings. Without patients’ prior knowledge of the role, or an understanding of how NPs functioned, establishing trusting relationships in all settings took time. Despite patients’ initial hesitation, once they were acquainted with the NP they were satisfied with their care and accepted the NP, as described by this participant:

"The patients took to [NP] quickly. And I don’t think we were really surprised at that. Once they realized [NP] could do everything a doctor could do and once they met and saw the extra time and care [NP] gave they were satisfied. Patients liked [NP’s] caring approach and that they didn’t feel rushed. [NP] explained things very well in terms they could understand. [NP] is very knowledgeable and that was obvious to them. [NP] knew what [NP] was talking about and they felt very confident in what [NP] told them."

Before the NP was hired, participants typically had a limited awareness or knowledge of the role, and acceptance of the NP within the team had to occur after the NP was hired. In PHC 1 and PHC 2, where the team knew the NP, acceptance of happened more quickly. Nonetheless, although team members may have been accepting of the idea of an NP, it was only after the NP was hired that acceptance of the individual NP occurred. Although we did not originally identify community members as stakeholders, it is clear that their involvement, knowledge and awareness of the NP role facilitated their acceptance, and acceptance of the role by all stakeholders was closely connected to their prior knowledge of the individual NP, and clarifying the intentions for the role.

### NP Role enactment

Implementing the NP role was influenced by how well others understood expectations for the role and their acceptance of the individual NP. Acceptance was influenced by prior knowledge of the NP and involvement in determining how the NP would function in the setting. In turn, the ability of the NP to actually carry out expectations and enact the role was influenced by how the role was implemented. We asked NPs to describe how they were enacting the role competencies of clinical practice, leadership, collaboration and change agent and research. Based on their descriptions we were able to determine that NPs were incorporating all of these competencies into their role.

**Figure 1:**
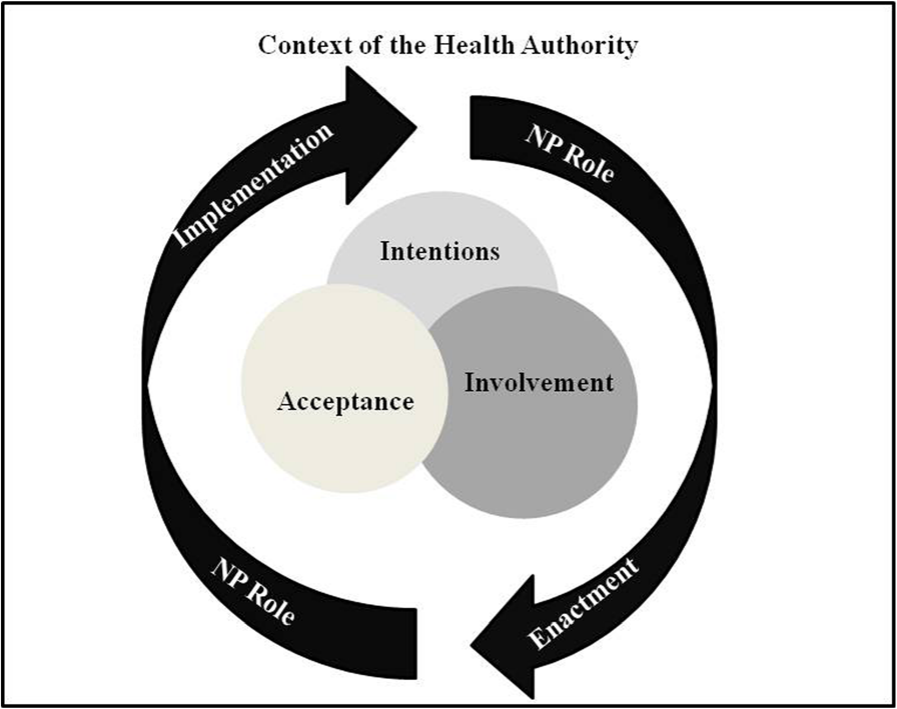
Context of the health Authority.

Based on the findings from this study, we developed a conceptual framework, Figure 1, indicating the interconnectedness of intention, involvement and acceptance and their influence the process of NP role implementation and NP role enactment. Although we identified the concepts from the literature, their relationship to the implementation process was unclear.

In this model the concepts of intention, involvement, and acceptance are interconnected indicating how they simultaneously influence role enactment and implementation. The concepts are situated with the context of the health authority in which the role was implemented. Through analysis we were able to determine that these concepts are interconnected and each is influenced by the other and all influence NP role implementation and enactment.

### Summary of key findings

In summary, we found that planning for the role beforehand and long-term planning after the NP was hired were important to help team members better understand the reason the NP role was implemented. Once the NP was hired team members needed to clarify the intentions for the role primarily because they were not involved earlier in the process and did not have a clear understanding of it. In the early stages of implementation, and immediately after the NP was hired, team members sought support and guidance from senior management within the health authority to clarify the intentions for the role. Unexpected changes in patient populations and/or in the context of the setting influenced how the NP would function. Acceptance of the NP was facilitated by team members’ prior knowledge of either the role or the individual. Community stakeholders who needed to or were expected to interact with the NP wanted to be involved in the implementation process and their acceptance of the NP developed as they gained knowledge and understanding of the role. Finally, although relatively new in their roles, approximately two years after being hired, NPs were enacting, to some degree, all competencies of the role as defined by CRNBC.

## Discussion

Implementation can be described as the transition period following a decision to adopt an innovation [41]. Context, environmental issues, and the characteristics of the individuals involved all influence implementation. Previous researchers identified barriers and facilitators to role implementation such as role clarity, team involvement, and planning for the role [2,10,11,26-28].

From the review of the literature we identified three sensitising concepts that influenced implementation: involvement, acceptance and intention. In this study we were able to verify that the three concepts influence implementation of the role and the ability of the NP to enact the role. Our findings also indicate the need to consider these three factors simultaneously throughout the implementation process, as demonstrated by the interconnectedness of the concepts in the conceptual framework, Figure 1. Without any one of the three concepts, not only is implementation difficult, but it makes it difficult for the NP to fulfill role expectations. In the early stages of implementation, when team members are just beginning to understand the role, it is important for as many of them as possible to be involved in discussing how the NP would function and defining the role. Equally important, team members need to be willing to accept the NP as a new member of the team.

### Contributions to knowledge

This study contributes to the state of the knowledge of role implementation in two ways. First, it explicates factors, such as intention, involvement, and acceptance, to consider when implementing new roles and highlights the importance of context. Second, it demonstrates the complexity of the role implementation process. Stakeholders need to expect the process to take time and to recognize that the process used in one setting might not work in another setting. This does not indicate that efforts in one setting were right and in the other setting they were wrong. Instead, the context of each setting will influence the process of implementation.

### Limitations

This was a single case study with three embedded units of analysis. The intent of the study was to understand and explain the process used to implement the NP role in the PHC settings of one health authority. Participants included only those who worked in PHC settings. As a result of focusing on the PHC setting, no data were collected from senior health authority managers. Therefore, perceptions of participants in PHC settings may not reflect organizational realities or the intent of senior health authority managers. As well, the perceptions of patients were not represented. Data from senior health authority managers could have provided insight into expectations for the NP role that were not conveyed by participants. Data from patients would have helped to explain how patients, seen by the NP, found the experience and their level of acceptance of the NP as a care provider.

We did not originally identify community members as stakeholders however; we learned that their involvement, knowledge and awareness of the NP role were important. We were only able to include one community member in the study. Additional community members would have added to our explanation of community members’ acceptance of NPs and their role in the implementation process.

Our findings are based on one interview with each of the 16 participants and documents obtained from each setting. Participant observations might have enhanced our understanding of team member interactions and NP role enactment. Another limitation is volunteer bias as only those participants who volunteered were interviewed. Others who chose not to volunteer might have had different perceptions.

All NP participants were novices when first hired into their positions. Our findings may not be transferable to settings where the NP is experienced, where participants are familiar with the NP role, or provinces where the role is better established within the health care system. Finally, we acknowledge that participants involved in implementing the NP role in the practice settings of other health authorities may have had different experiences.

### Implications

This study contributes to an in-depth understanding of the process of implementing new health care roles. It demonstrates the need for policy makers and other stakeholders to consider multiple factors when implementing the NP role in unfamiliar settings. We identified that citizen engagement was inadequate in efforts to implement the NP role in the health authority. As we move from a disease-centred to a patient-centred approach to PHC (World Health Organization, 2008) it becomes increasingly necessary to hear directly from consumers and to have them fully engaged in legitimate partnerships [42] with policy makers and health authorities in determining how and where to implement NP roles.

These findings need to be built upon to determine what other factors may influence NP role implementation. Our explanation of the complexity of implementing the role was limited to the practice settings; there remains a need to research factors influencing implementation at a systems and organizational level.

## Conclusion

This study enriches our understandings of how clearly identified intentions for the NP role and the involvement of key stakeholders can influence acceptance of the role and the process of role implementation. It helps to explain how these factors influence the ability of the NP to enact fully all the advanced nursing practice competencies as set out by CRNBC. The role is new in many countries and these findings are relevant internationally because they emphasize the pivotal role of managers in successful implementation. The findings suggest that managers need to attend to how others are involved in the process, how the role is defined, and the degree of acceptance for the role. Without strong organizational leadership, this new role, just like any new innovation, is at risk of failure because it is not taken up by the practice settings.

## Competing interests

The authors declare they have no competing interests.

## Authors’ contributions

All authors were involved in the design of the research study. ESG conceptualized the research questions and analytic approach for this manuscript. Analyses were conducted by ESG with support from RMM and FB. ESG contributed to the majority of the writing, all authors contributed to the final editing and approval of the manuscript.

## Pre-publication history

The pre-publication history for this paper can be accessed here:

http://www.biomedcentral.com/1472-6955/12/1/prepub
